# Supplementary material for: Maternal aryl hydrocarbon receptor activation protects newborns against necrotizing enterocolitis
Source: Nat Commun. 2021 Feb 15;12:1042. doi: 10.1038/s41467-021-21356-4 (PMC7884836; doi:10.1038/s41467-021-21356-4)
Supplement: Supplementary file 1 — Supplementary Information [file 41467_2021_21356_MOESM1_ESM.pdf]

Supplementary Fig. 1, Lu et al.

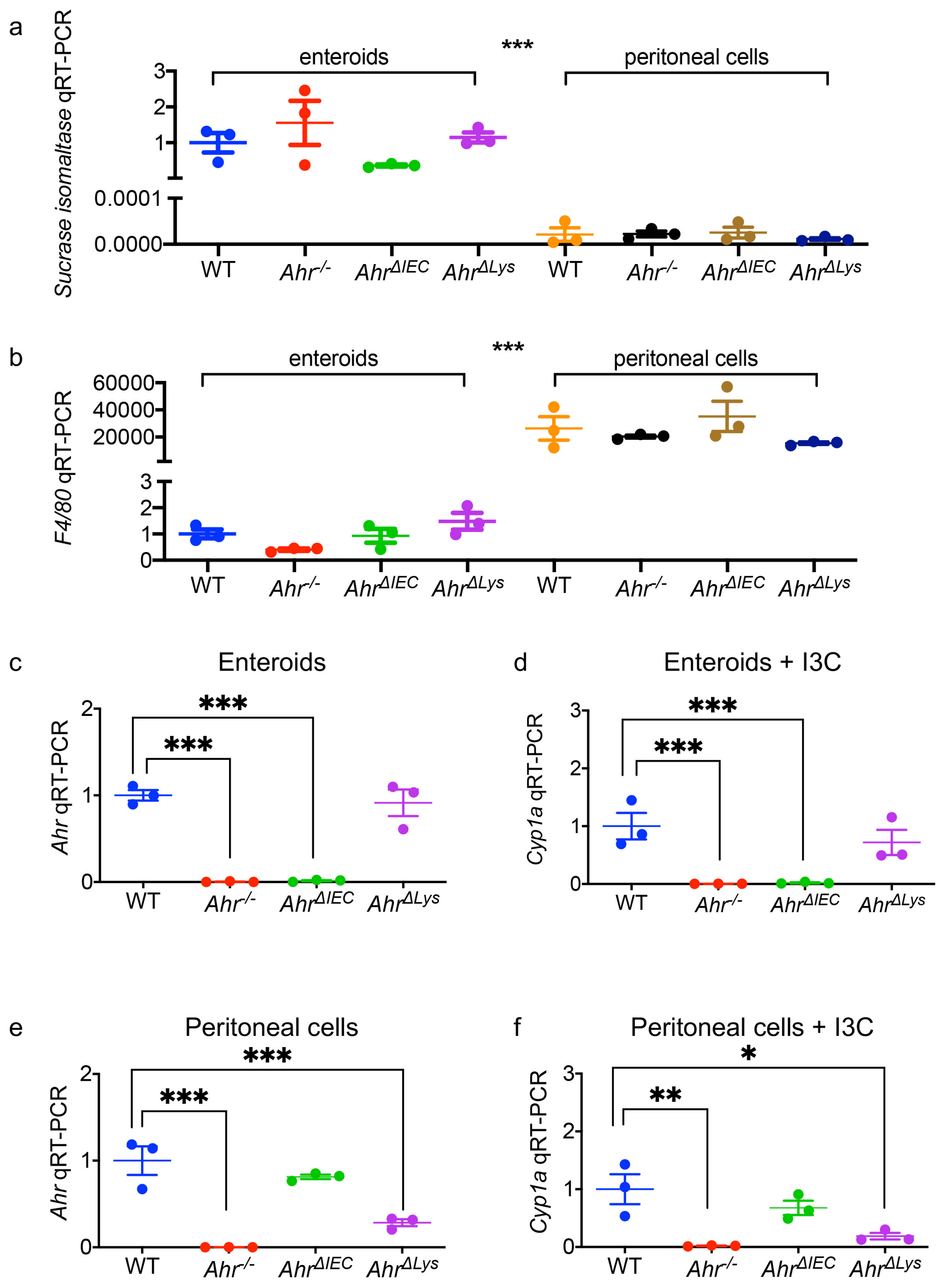

Supplementary Fig. 1. *Generation of Ahr conditional knockout mice.* **a-b**, qRT-PCR showing expression of epithelial cell marker *sucrase isomaltase* **(a**, n=3, 3, 3, 3 enteroids from individual mouse and 3, 3, 3, 3 peritoneal cells from individual mouse, enteroids vs peritoneal cells p<0.0001**)** and macrophage marker *F4/80* **(b**, n=3, 3, 3, 3 enteroids from individual mouse and 3, 3, 3, 3 peritoneal cells from individual mouse, enteroids vs peritoneal cells p<0.0001**)** in the enteroids and peritoneal cells from p11 wild-type (WT), $Ahr^{-/-}$, Ahr^{DIEC} and Ahr^{Dlys} mice. **c**, qRT-PCR showing the expression of *Ahr* in the enteroids (n=3, 3, 3, 3 enteroids from individual mouse, WT vs $Ahr^{-/-}$ p<0.0001, WT vs Ahr^{DIEC} p<0.0001). **d**, qRT-PCR showing expression of *Cyp1a1* in the enteroids treated with I3C (200mM overnight) (n=3, 3, 3, 3 enteroids from individual mouse, WT vs $Ahr^{-/-}$ p=0.0055, WT vs Ahr^{DIEC} p=0.0060). **e**, qRT-PCR showing expression of *Ahr* in the peritoneal cells (n=3, 3, 3, 3 peritoneal cells from individual mouse, WT vs $Ahr^{-/-}$ p=0.0001, WT vs Ahr^{DLys} p=0.0009). **f**, qRT-PCR showing expression of *Cyp1a1* in the peritoneal cells treated with I3C (200mM overnight) (n=3, 3, 3, 3 peritoneal cells from individual mouse, WT vs $Ahr^{-/-}$ p=0.0038, WT vs Ahr^{DLys} p=0.0111). All data are presented as mean values +/- SEM. *$p < 0.05$, **$p < 0.01$, ***$p < 0.001$, p values obtained either from two-sided t-tests or using one-way ANOVA followed by multiple comparisons. Each dot in graphs represents data from an individual mouse.

Supplementary Fig. 2, Lu et al.

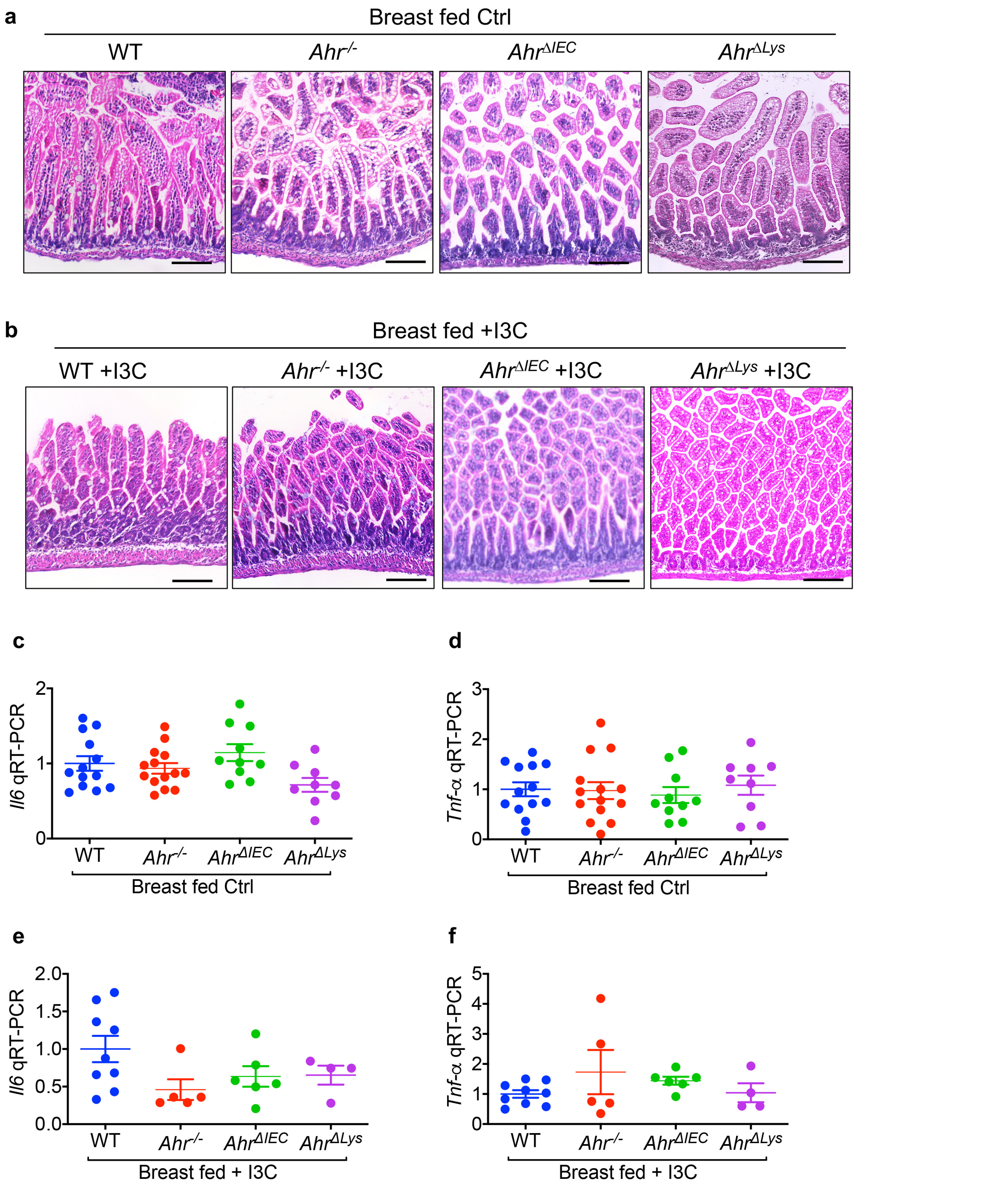

Supplementary Fig. 2. *Evaluation of Ahr transgenic mice treated with I3C.* **a-b,** representative images of H&E-stained ileal sections from breast fed and/or I3C-treated (25mg per kg body weight per day for 4 days) newborn wild-type, *Ahr*$^{-/-}$, *Ahr*^{DIEC} and *Ahr*^{Dlys} mice. **c-f**, qRT-PCR showing expression of *Il6* (**c**, n=13, 14, 10, 9 mice, **e**, n=9, 5, 6, 4 mice) and *Tnf-a* (**d**, n=13, 14, 10, 9 mice, **f**, n=9, 5, 6, 4 mice) under the indicated conditions. Scale bars in **a**,**b**, 100µm. All data are presented as mean values +/- SEM. Each dot represents data from an individual mouse.

Supplementary Fig. 3, Lu et al.

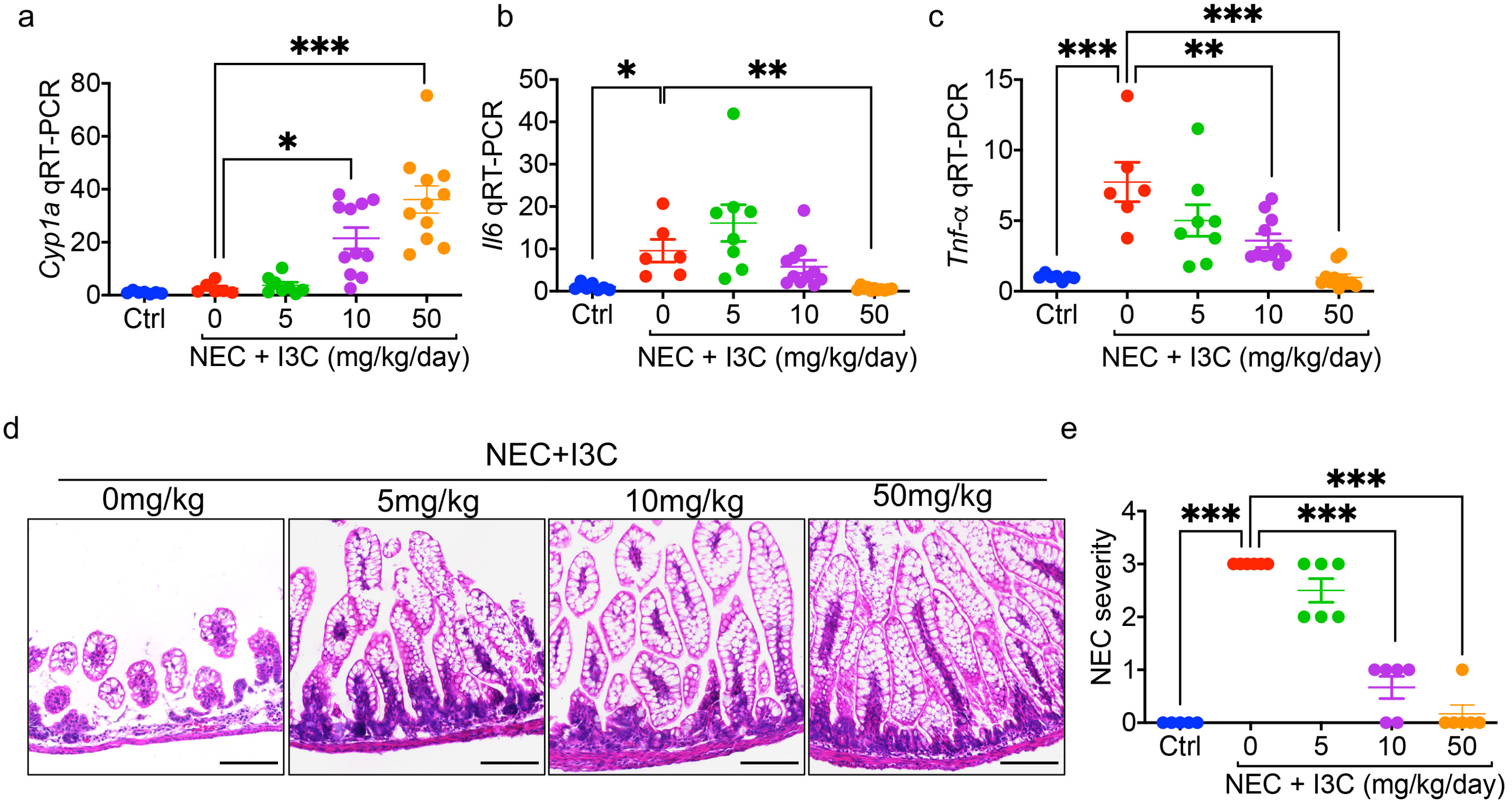

Supplementary Fig. 3. *Dose-response of I3C in NEC.* **a-c**, qRT-PCR showing mRNA expression of *Cyp1a1* (**a**, n=7, 6, 8, 11, 11 mice, 0 vs 10 p=0.0237, 0mg per kg body weight per day vs 50mg per kg body weight per day p<0.0001), *Il6* (**b**, n=7, 6, 8, 11, 11 mice, Ctrl vs NEC p=0.0054, NEC vs NEC +I3C 50mg per kg body weight per day p=0.0003) and *Tnf-a* (**c**, n=7, 6, 8, 11, 11 mice, Ctrl vs NEC p<0.0001, NEC vs NEC +I3C 10mg per kg body weight per day p=0.0026, NEC vs NEC +I3C 50mg per kg body weight per day p<0.0001) in the ileum of wild-type mice subjected to experimental NEC without or with I3C (5mg per kg body weight per day, 10mg per kg body weight per day, 50mg per kg body weight per day for 4 days). **d**, representative H&E-stained ileum. **e**, NEC severity (n=6, 6, 6, 6, 6 mice, Ctrl vs NEC p<0.0001, NEC vs NEC +I3C 10mg per kg body weight per day p=0.0026, NEC vs NEC +I3C 50mg per kg body weight per day p<0.0001). Scale bars in **d**, 100µm. All data are presented as mean values +/- SEM. *$p < 0.05$, **$p < 0.01$, ***$p < 0.001$, p values obtained from two-sided t-tests or one-way ANOVA followed by multiple comparisons. Each dot in graphs represents data from an individual mouse.

Supplementary Fig. 4, Lu et al.

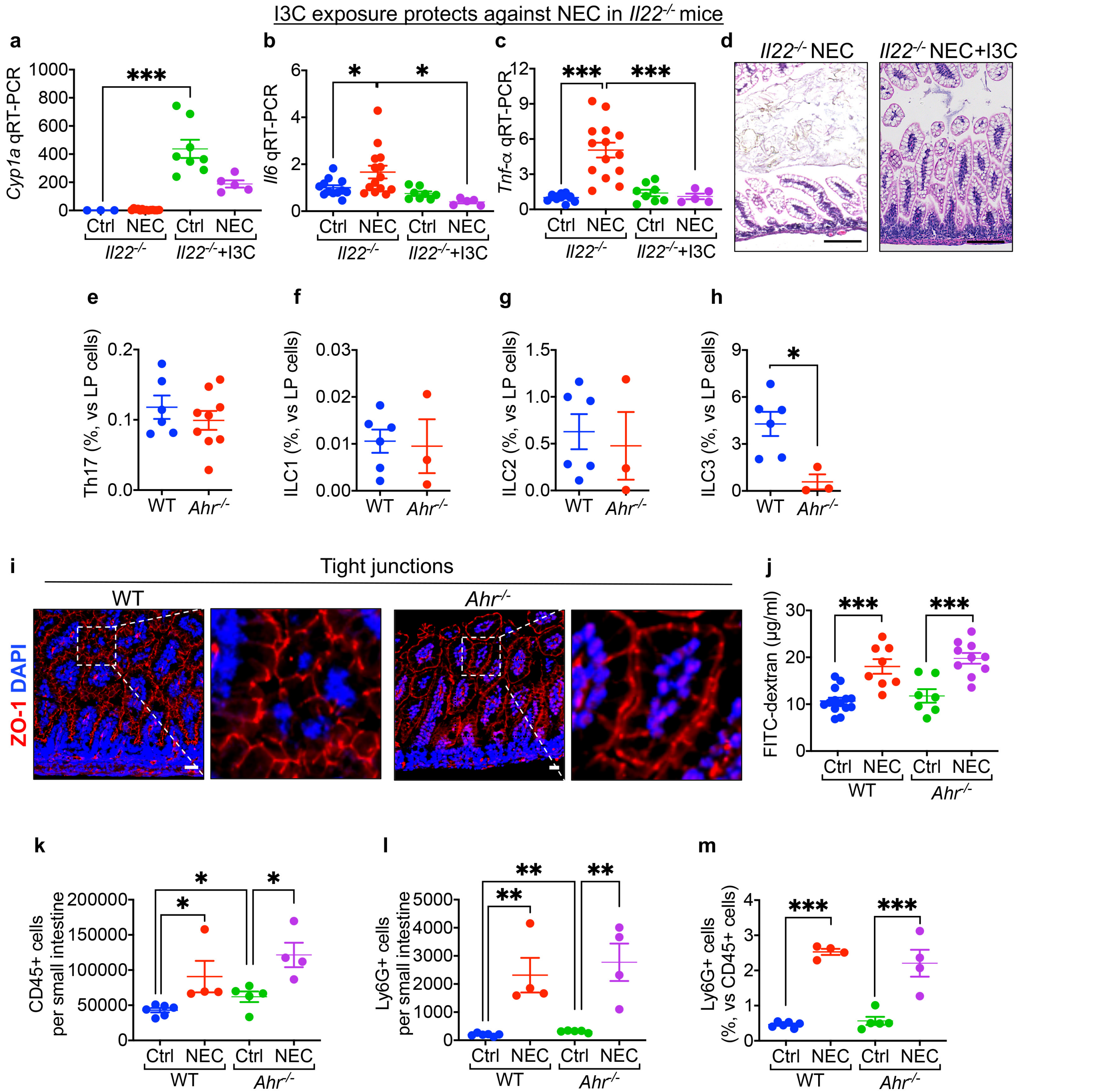

Supplementary Fig. 4. *Ahr activation protects against NEC independent of IL-22, intestinal permeability, CD45 cells and neutrophils.* **a-d**, qRT-PCR showing mRNA expression of *Cyp1a1* (**a**, n=3, 14, 8, 5 mice, Ctrl $Il22^{-/-}$ vs Ctrl $Il22^{-/}$ +I3C, p<0.0001), *Il6* (**b**, n=12, 14, 8, 5 mice, CTRL $Il22^{-/-}$ vs NEC $Il22^{-/-}$ p=0.0413, NEC $Il22^{-/-}$ vs NEC $Il22^{-/-}$ +I3C p=0.0137) and *Tnf-a* (**c**, n=12, 14, 8, 5 mice, CTRL $Il22^{-/-}$ vs NEC $Il22^{-/-}$ p<0.0001, NEC $Il22^{-/-}$ vs NEC $Il22^{-/}$ +I3C p<0.0001), and representative H&E-stained images (**d**) of the ileum of $Il22^{-/-}$ mice subjected to experimental NEC without or with I3C (+I3C, 25mg per kg body weight per day for 4 days). **e-h**, flow cytometry showing % relative to total lamina propria (LP) cells of Th17 cells (**e**, n=6, 9 mice), ILC1 cells (**f**, n=6, 3 mice), ILC2 cells (**g**, n=6, 3 mice) and ILC3 cells (**h**, n=6, 3 mice, p=0.0160) in the lamina propria of ileum of WT and $Ahr^{-/-}$ mice. **i**, representative confocal images stained with ZO-1 in the ileum of WT and $Ahr^{-/-}$ mice. ZO-1, red singal; nuclei (DAPI, blue signal). **j**, concentration of 4-kDa FITC-dextran in the serum of WT and $Ahr^{-/-}$ mice without and with NEC showing the intestinal permeability (n=14, 8, 7, 10 mice, WT Ctrl vs WT NEC p=0.0002, $Ahr^{-/-}$ Ctrl vs $Ahr^{-/-}$ NEC p=0.0003). **k-m**, the number of CD45+ cells (**k**, n=6, 4, 5, 4 mice, WT Ctrl vs WT NEC p=0.0303, WT Ctrl vs $Ahr^{-/-}$ Ctrl p=0.0348, $Ahr^{-/-}$ Ctrl vs $Ahr^{-/-}$ NEC p=0.0117), the number of Ly6G+ neutrophis (**l**, n=6, 4, 5, 4 mice, WT Ctrl vs WT NEC p=0.0024, WT Ctrl vs $Ahr^{-/-}$ Ctrl p=0.0017, $Ahr^{-/-}$ Ctrl vs $Ahr^{-/-}$ NEC p=0.0040) and the percentage of neutrophils in CD45+ cells (**m**, n=6, 4, 5, 4 mice, WT Ctrl vs WT NEC p<0.0001, $Ahr^{-/-}$ Ctrl vs $Ahr^{-/-}$ NEC p<0.0001) in the lamina propria of WT and $Ahr^{-/-}$ mice without and with NEC. Scale bars in **d**, 100 μm. Scale bars in **i**, 25 μm. All data are presented as mean values +/- SEM. *$p < 0.05$, **$p < 0.01$, ***$p < 0.001$, p values obtained from two-sided *t*-tests or one-way ANOVA followed by multiple comparisons. Each dot in graphs represents data from an individual mouse.

Supplementary Fig. 5, Lu et al.

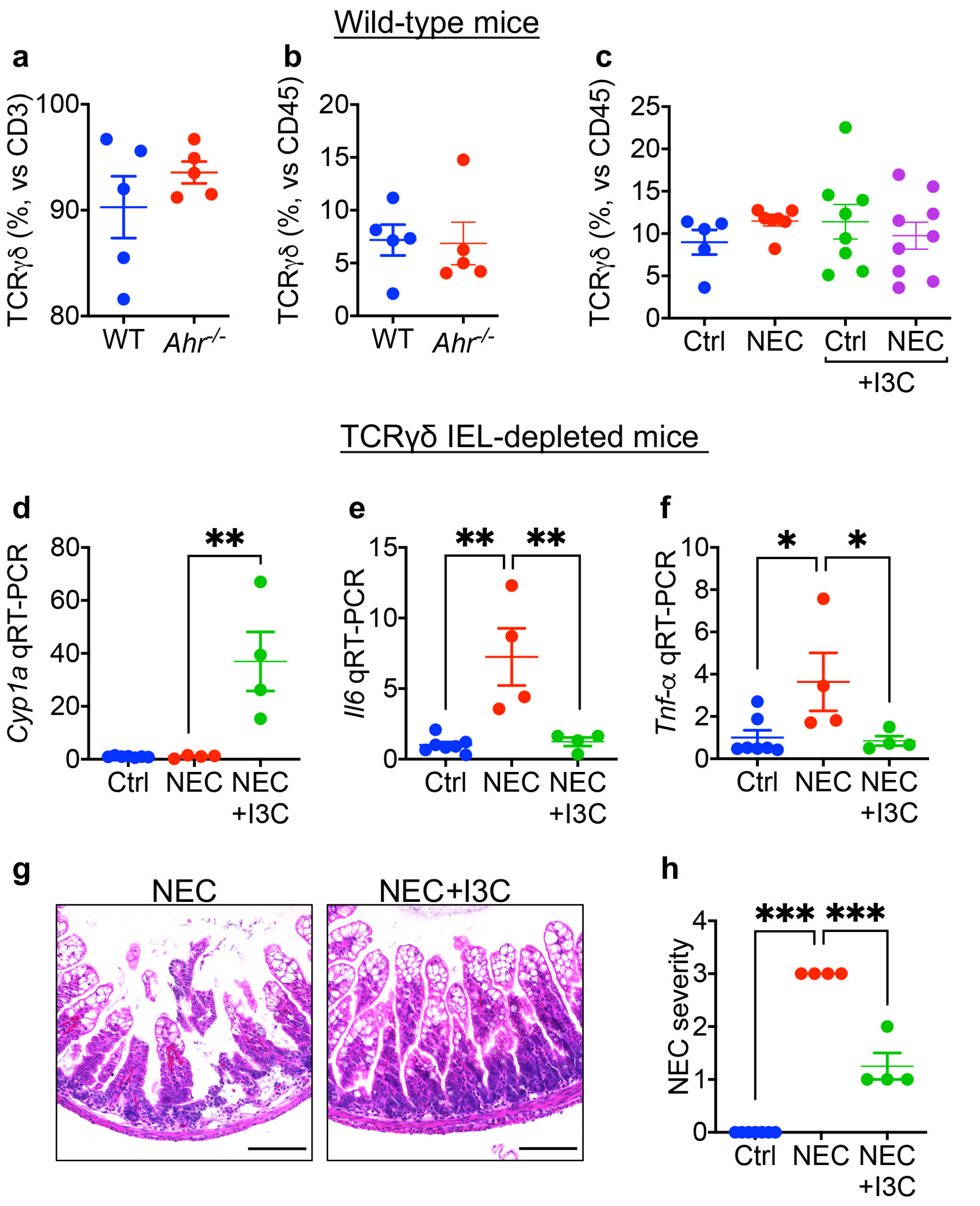

Supplementary Fig. 5. *Ahr activation protects against NEC independent of IELs.* **a-b**, flow cytometry showing the percentage of TCRγδ T cells of CD3 T cells (**a**, n=5, 5 mice) and the percentage of TCRγδ T cells of CD45 IEL cells (**b**, n=5, 5 mice) in the small intestine of wild-type (WT) and *Ahr*$^{-/-}$ mice. **c**, the percentage of TCRγδ T cells of CD45 IEL cells in the small intestine of wild-type mice subjected to experimental NEC without or with I3C (+I3C, 25mg per kg body weight per day for 4 days) (n=5, 7, 8, 9 mice). **d-h**, qRT-PCR showing mRNA expression of *Cyp1a1* (**d**, n=7, 4, 4 mice, NEC vs NEC +I3C p=0.0018), *Il6* (**e**, n=7, 4, 4 mice, Ctrl vs NEC p=0.0012, NEC vs NEC +I3C p=0.0041) and *Tnf-a* (**f**, n=7, 4, 4 mice, Ctrl vs NEC p=0.0318, NEC vs NEC +I3C p=0.0431), representative H&E-stained images (**g**), and NEC severity (**h**, n=7, 4, 4 mice, Ctrl vs NEC p<0.0001, NEC vs NEC +I3C p<0.0001) of the in the ileum of TCRγδ T IEL-depleted mice subjected to experimental NEC without or with I3C (+I3C, 25mg per kg body weight per day for 4 days). Scale bars in **g**, 100 µm. All data are presented as mean values +/- SEM. *p < 0.05, **p < 0.01, ***p < 0.001, p values obtained from two-sided t-tests or one-way ANOVA followed by multiple comparisons. Each dot in graphs represents data from an individual mouse.

Supplementary Fig. 6, Lu et al.

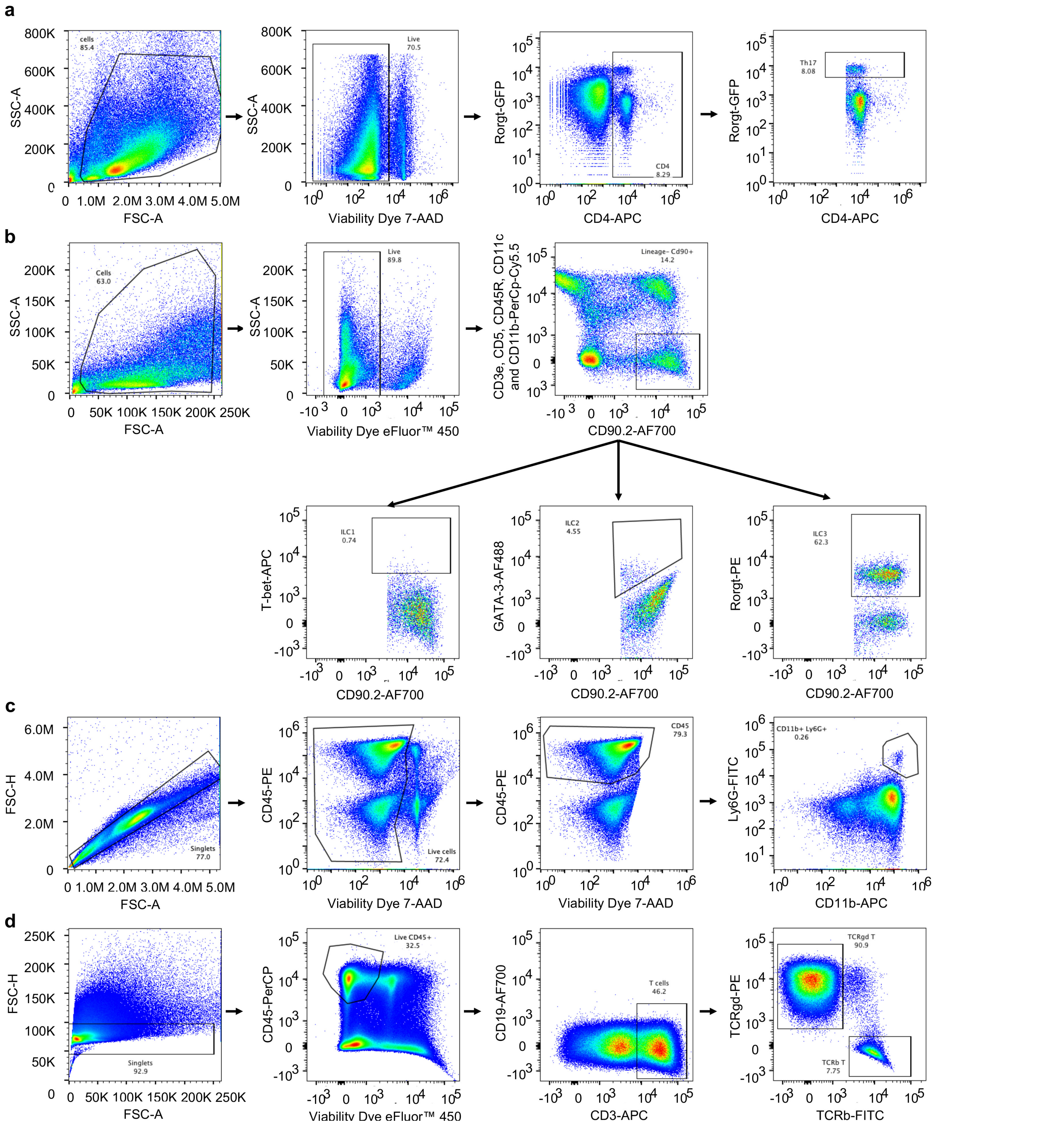

Supplementary Fig. 6. *Gating strategies for flow cytometry analysis*. **a**, for Th17 cells (Supplementary Fig. 4e), LP cells (SSC-A vs. FSC-A) were first gated for the uptake of the Live/Dead stain to determine live versus dead cells, and the live cell gate was further analyzed for the expression of CD4 and Rorg-GFP. **b**, for ILCs (Supplementary Fig. 4f-h), LP cells (SSC-A vs. FSC-A) were first gated for the uptake of the Live/Dead stain to determine live versus dead cells, and the live cell gate was further analyzed for the expression of hematopoietic cell lineages (CD3e, CD5, CD45R, CD11c and CD11b) and CD90.2. Then the expression of T-bet (ILC1), GATA-3 (ILC2) and RORgt (ILC3) was determined from the lineages- CD90+ gate. **c**, for CD45+ cells and neutrophils (Supplementary Fig. 4k-m), LP cells were first gated for singlets (FSC-H vs. FSC-A) and for the uptake of the Live/Dead stain to determine live versus dead cells. The live cell gate was further analyzed for the expression of CD45. Then the expression of CD11b and Ly6G was determined from the CD45+ gate. **d**, for IELs (Supplementary Fig. 5a-c), the IEL cells (SSC-A vs. FSC-A) were first gated for singlets (FSC-H vs. FSC-A), and then for the uptake of the Live/Dead stain to determine live versus dead cells and the expression of CD45. The CD45+ gate was furthere analyzed for CD19 and CD3, and finally the CD19- CD3+ gate was analyzed for TCRgd and TCRb expression.

Supplementary Table 1. Primers for mouse genotyping.

Target	Genotyping primer sequence
Ahr$^{-/-}$	GTCACTCAGCATTACACTTTCTA, GGTACAAGTGCACATGCCTGC (Knockout:180bp)
Ahr^{fx}	CAGTGGGAATAAGGCAAGAGTGA, GGTACAAGTGCACATGCCTGC (Wild type:106bp, loxP:140bp)
Cre	GTTCGCAAGAACCTGATGGACA, CTAGAGCCTGTTTTGCACGTTC (Transgene:339bp)
IL22$^{-/-}$	CAGGCTCTCCTCTCAGTTATCA, TCCTGAAGGCCAAAATAGG, CCTCAGGTTCAGCAG GGAAC (Wild type:424bp, mutant:313bp)
RORγt^{GFP}	CCCCCTGCCCAGAAACACT, GGATGCCCCCATTCACTTACTTCT, CGGACACGCTGA ACTTGTGG (Wild type:174bp, mutant:241bp)
TCRd^{creERT2}	ACACCGGCCTTATTCCAAG, GGAGAGTTTTCCTAGCAGCA, GCTTCCAAAACACTTGCACA (Wild type:312bp, mutant:250bp)
ROSA^{iDTR}	CATCAAGGAAACCCTGGACTACTG, AAAGTCGCTCTGAGTTGTTAT, GGAGCGGGAGAAATGGATATG (Wild type:603bp, mutant:242bp)

Supplementary Table 2. *qRT-PCR primers for mouse, human, pig and rat genes.*

Gene	Forward primer sequence	Reverse primer sequence	Amplicon size (bp)
Mouse gene			
Ahr	ATGTCCATGTATCAGTGCCAG	CTGCTCAAGTCGGACGAATAG	149
IL6	CCAATTTCCAATGCTCTCCT	ACCACAGTGAGGAATGTCCA	182
Rplp0	GGCGACCTGGAAGTCCAACT	CCATCAGCACCACAGCCTTC	143
Tlr4	TTTATTCAGAGCCGTTGGTG	CAGAGGATTGTCCTCCCATT	186
Tnf-α	TTCCGAATTCACTGGAGCCTCGAA	TGCACCTCAGGGAAGAATCTGGAA	144
F4/80	GCTCCTGGGTGCTGGGCATT	TCCCGTACCTGACGGTTGAGCA	133
Sucrase isomaltase	GCCCATATTCATGGTGGAAC	TCCAATGACAGGAGTCACCA	126
Human gene			
AHR	GTCCAGTCTAATGCACGCCT	ATGGCAGGAAAAGGGTTGGT	182
CYP1A1	AATTTCGGGGAGGTGGTTGG	GATGTGGCCCTTCTCAAAGGT	168
RPLP0	GGCGACCTGGAAGTCCAACT	CCATCAGCACCACAGCCTTC	143
TNF-α	GGCGTGGAGCTGAGAGATAAC	GGTGTGGGTGAGGAGCACAT	120
Pig gene			
Ahr	CCACTTCAGCCACCATCCAT	ATGCACAGCTCTGCTTCAGT	140
Rplp0	GGCGACCTGGAAGTCCAACT	CCATCAGCACCACAGCCTTC	143
Rat gene			
Cyp1a1	TCCTGGAGACCTTCCGACAT	AACCTGCCACTGGTTCACAA	128
Rplp0	GGCGACCTGGAAGTCCAACT	CCATCAGCACCACAGCCTTC	143

Supplementary Table 3. *Primers for mouse microRNAs.*

microRNA	Primer sequence
let-7i	TGAGGTAGTAGTTTGTGCTGTT
miR-146b	TGAGAACTGAATTCCATAGGCT
miR-223	TGTCAGTTTGTCAAATACCCCA
miR-191	CAACGGAATCCCAAAAGCAGCTG
