# Supplementary material for: Maternal aryl hydrocarbon receptor activation protects newborns against necrotizing enterocolitis
Source: Nat Commun. 2021 Feb 15;12:1042. doi: 10.1038/s41467-021-21356-4 (PMC7884836; doi:10.1038/s41467-021-21356-4)
Supplement: Supplementary file 3 — Reporting Summary [file 41467_2021_21356_MOESM3_ESM.pdf]

nature research

Corresponding author(s): David J. Hackam & Peng Lu

Last updated by author(s): 01/25/2021

Reporting Summary

Nature Research wishes to improve the reproducibility of the work that we publish. This form provides structure for consistency and transparency in reporting. For further information on Nature Research policies, see Authors & Referees and the Editorial Policy Checklist .

Statistics

For all statistical analyses, confirm that the following items are present in the figure legend, table legend, main text, or Methods section.

n/a	Confirmed	
☐	☒	The exact sample size (*n*) for each experimental group/condition, given as a discrete number and unit of measurement
☐	☒	A statement on whether measurements were taken from distinct samples or whether the same sample was measured repeatedly
☐	☒	The statistical test(s) used AND whether they are one- or two-sided<br>*Only common tests should be described solely by name; describe more complex techniques in the Methods section.*
☒	☐	A description of all covariates tested
☒	☐	A description of any assumptions or corrections, such as tests of normality and adjustment for multiple comparisons
☐	☒	A full description of the statistical parameters including central tendency (e.g. means) or other basic estimates (e.g. regression coefficient) AND variation (e.g. standard deviation) or associated estimates of uncertainty (e.g. confidence intervals)
☐	☒	For null hypothesis testing, the test statistic (e.g. *F*, *t*, *r*) with confidence intervals, effect sizes, degrees of freedom and *P* value noted<br>*Give P values as exact values whenever suitable.*
☒	☐	For Bayesian analysis, information on the choice of priors and Markov chain Monte Carlo settings
☒	☐	For hierarchical and complex designs, identification of the appropriate level for tests and full reporting of outcomes
☒	☐	Estimates of effect sizes (e.g. Cohen's *d*, Pearson's *r*), indicating how they were calculated

Our web collection on statistics for biologists contains articles on many of the points above.

Software and code

Policy information about availability of computer code

Data collection	FACSDiva v8.0.2 software was used to collect data from BD LSRII flow cytometer.<br>BD CSampler v1.0.264.21 Software was used to collect data from BD Accuri™ C6 Plus flow cytometer.<br>SoftMax Pro v6.4.2 Software was used to collect data from the luciferase assay and FITC-dextron assay.<br>Bio-Rad CFX Manager 3.1 software was used to collect data from qRT-PCR.<br>Leica Application Suite X v3.4.2.18368 software was used to take images for H&E staining.<br>NIS-Elements AR v4.10.01 software was used to take images for IF staining.
Data analysis	Prism 9, analysis and graphing software from GraphPad.<br>FlowJo™ v10.6.1, flow cytometry analysis and graphing software from FlowJo.

For manuscripts utilizing custom algorithms or software that are central to the research but not yet described in published literature, software must be made available to editors/reviewers. We strongly encourage code deposition in a community repository (e.g. GitHub). See the Nature Research guidelines for submitting code & software for further information.

Data

Policy information about availability of data

All manuscripts must include a data availability statement. This statement should provide the following information, where applicable:

- Accession codes, unique identifiers, or web links for publicly available datasets
- A list of figures that have associated raw data
- A description of any restrictions on data availability

All relevant data are available from the corresponding authors upon reasonable request. Source data are provided with this paper.

Field-specific reporting

Please select the one below that is the best fit for your research. If you are not sure, read the appropriate sections before making your selection.

- [x] Life sciences
- [ ] Behavioural & social sciences
- [ ] Ecological, evolutionary & environmental sciences

For a reference copy of the document with all sections, see nature.com/documents/nr-reporting-summary-flat.pdf

Life sciences study design

All studies must disclose on these points even when the disclosure is negative.

Sample size	The sample size was determined using power analysis.
Data exclusions	No data exclusions from the analysis.
Replication	All experiments were repeated three times with similar results.
Randomization	All experiments were randomly assigned into experimental groups.
Blinding	The investigators were blinded to group allocation during the sample collection and analysis.

Reporting for specific materials, systems and methods

We require information from authors about some types of materials, experimental systems and methods used in many studies. Here, indicate whether each material, system or method listed is relevant to your study. If you are not sure if a list item applies to your research, read the appropriate section before selecting a response.

Materials & experimental systems

n/a	Involved in the study	
[ ]	[x]	Antibodies
[ ]	[x]	Eukaryotic cell lines
[x]	[ ]	Palaeontology
[ ]	[x]	Animals and other organisms
[ ]	[x]	Human research participants
[x]	[ ]	Clinical data

Methods

n/a	Involved in the study	
[x]	[ ]	ChIP-seq
[ ]	[x]	Flow cytometry
[x]	[ ]	MRI-based neuroimaging

Antibodies

Antibodies used

Antibodies for immunofluorescence staining are goat-anti AHR (clone M-20, Catalog # sc-8089, Santa Cruz), goat-anti Ecadherin (Catalog # AF748, R&D Systems), rabbit-anti ZO-1 (Catalog # 40-2200, Thermo Fisher Scientific), and mouse-anti NF-κB p65 (clone F-6, Catalog # sc-8008, Santa Cruz).

Antibodies for flow cytometry are rat anti-CD16/CD32 (clone 93, Catalog # 101320, BioLegend), rat anti-mouse CD90.2 Alexa Fluor® 700 (clone 30-H12, Catalog # 105319, BioLegend), armenian hamster anti-mouse CD3e PerCP-Cyanine5.5 (clone 145-2C11, Catalog # 16-0031-81, eBioscience), rat anti-mouse CD5 PerCP-Cyanine5.5 (clone 53-7.3, Catalog # 45-0051-80, eBioscience), rat anti-mouse CD45R (B220) PerCP-Cyanine5.5 (clone RA3-6B2, Catalog # 45-0452-80, eBioscience), armenian hamster anti-mouse CD11c PerCP-Cyanine5.5 (clone N-418, Catalog # 45-0114-80, eBioscience), rat anti-mouse CD11b PerCP-Cyanine5.5 clone (M1/70, Catalog # 45-0112-80, eBioscience), rat anti-mouse Gata-3 PE-Cyanine7 (clone TWAJ, Catalog # 25-9966-41, eBioscience), mouse anti-mouse T-bet eFluor® 660 (clone 4B10, Catalog # 50-5825-80, eBioscience), rat anti-mouse ROR gamma (t) PE (clone B2D, Catalog # 12-6981-80, eBioscience), rat anti-mouse EOMES Alexa Fluor® 488 (clone Dan11mag, Catalog # 53-4875-80, eBioscience), rat anti-mouse CD4 APC (clone RM4-5, Catalog # 561091, BD Biosciences), rat anti-mouse CD45 PerCP-Cyanine5.5 (clone I3/2.3, Catalog # 147705, BioLegend), rat anti-mouse CD19 Alexa Fluor® 700 (clone 1D3, Catalog # 56-0193-80, eBioscience), rat anti-mouse CD3 APC (clone 17A2, Catalog # 17-0032-80, eBioscience), armenian hamster anti-mouse TCR gamma/delta PE (clone GL-3, Catalog # 12-5711-81, eBioscience), armenian hamster anti-mouse TCR beta FITC (clone H57-597, Catalog # 11-5961-81, eBioscience), rat anti-mouse CD45 PE (clone I3/2.3, Catalog # 147711, BioLegend), rat anti-mouse CD11b APC (clone M1/70, Catalog # 101211, BioLegend), and rat anti-mouse Ly6G FITC (clone 1A8-Ly6g, Catalog # 11-9668-80, eBioscience).

Validation

Goat-anti AHR (clone M-20, Catalog # sc-8089, Santa Cruz), immunofluorescence staining, validation information from manufacturer's website. This antibody has 9 citations.

Goat-anti Ecadherin (Catalog # AF748, R&D Systems), immunofluorescence staining, validation information from manufacturer's website. E-Cadherin was detected in immersion fixed mouse intestinal organoids using Goat Anti-Human/Mouse E-Cadherin

Antigen Affinity-purified Polyclonal Antibody (Catalog # AF748) at 10 µg/mL for 3 hours at room temperature.

Rabbit-anti ZO-1 (Catalog # 40-2200, Thermo Fisher Scientific), immunofluorescence staining, validation information from manufacturer's website. Validated using MDCKII cells, Caco-2 cells, mouse brain and liver tissues. This Antibody was verified by Knockdown to ensure that the antibody binds to the antigen stated.

Mouse-anti NF-κB p65 (clone F-6, Catalog # sc-8008, Santa Cruz), immunofluorescence staining, validation information from manufacturer's website. Validated using SW480 cells showing nuclear and cytoplasmic localization. This antibody has 1589 citations.

Rat anti-CD16/CD32 (clone 93, Catalog # 101320, BioLegend), flow cytometry, validation information from manufacturer's website. This antibody has 195 citations.

Rat anti-mouse CD90.2 Alexa Fluor® 700 (clone 30-H12, Catalog # 105319, BioLegend), flow cytometry, validation information from manufacturer's website. Validated using C57BL/6 mouse thymocytes stained with 30-H12 Alexa Fluor® 700. This antibody has 12 citations.

Armenian hamster anti-mouse CD3e PerCP-Cyanine5.5 (clone 145-2C11, Catalog # 16-0031-81, eBioscience), flow cytometry, validation information from manufacturer's website. Validated using mouse splenocytes with Anti-Mouse CD3e FITC or PE (appropriate isotype controls were used). This antibody has 80 citations for flow cytometry application.

Rat anti-mouse CD5 PerCP-Cyanine5.5 (clone 53-7.3, Catalog # 45-0051-80, eBioscience), flow cytometry, validation information from manufacturer's website. Validated using C57BL/6 splenocytes with Anti-Human/Mouse CD45R (B220) APC (Product # 17-0452-82) and 0.06 µg of Rat IgG2a K Isotype Control PerCP-Cyanine5-5 (Product # 45-4321-80) or 0.06 µg of Anti-Mouse CD5 PerCP-Cyanine5-5. This antibody has 16 citations for flow cytometry application.

Rat anti-mouse CD45R (B220) PerCP-Cyanine5.5 (clone RA3-6B2, Catalog # 45-0452-80, eBioscience), flow cytometry, validation information from manufacturer's website. Validated using C57Bl/6 splenocytes with Anti-Mouse CD3 eFluor® 450 (Product # 48-0032-82) and 0.25 µg of Rat IgG2a K Isotype Control PerCP-Cyanine5-5 (Product # 45-4321-80) or 0.25 µg of Anti-Human/ Mouse CD45R (B220) PerCP-Cyanine5-5. This antibody has 84 citations for flow cytometry application.

Armenian hamster anti-mouse CD11c PerCP-Cyanine5.5 (clone N-418, Catalog # 45-0114-80, eBioscience), flow cytometry, validation information from manufacturer's website. Validated using C57BL/6 splenocytes with Anti-Human/Mouse CD45R (B220) APC (Product # 17-0452-82) and 0.125 µg of Armenian Hamster IgG Isotype Control PerCP-Cyanine5-5 (Product # 45-4888-80) or 0.125 µg of Anti-Mouse CD11c PerCP-Cyanine5-5. This antibody has 72 citations for flow cytometry application.

Rat anti-mouse CD11b PerCP-Cyanine5.5 (clone M1/70, Catalog # 45-0112-80, eBioscience), flow cytometry, validation information from manufacturer's website. Validated using mouse bone marrow cells with 0.125 µg of Rat IgG2b K Isotype Control PerCP-Cyanine5-5 (Product # 45-4031-80) or 0.125 µg of Anti-Mouse CD11b PerCP-Cyanine5-5. This antibody has 103 citations for flow cytometry application.

Rat anti-mouse Gata-3 PE-Cyanine7 (clone TWAJ, Catalog # 25-9966-41, eBioscience), flow cytometry, validation information from manufacturer's website. Validated using C57Bl/6 thymocytes with Anti-Mouse CD4 FITC (Product # 11-0042-82) and Anti-Mouse CD8a APC (Product # 17-0081-82) followed by intracellular staining with Rat IgG2b K Isotype Control PE-Cyanine7 (Product # 25-4031-82) or Anti-Human/Mouse Gata-3 PE-Cyanine7. This antibody has 10 citations for flow cytometry application.

Mouse anti-mouse T-bet eFluor® 660 (clone 4B10, Catalog # 50-5825-80, eBioscience), flow cytometry, validation information from manufacturer's website. Validated using normal human peripheral blood cells with Anti-Human CD8a FITC (Product # 11-0088-42) and 0.25 µg of Mouse IgG1 K Isotype Control eFluor® 660 (Product # 50-4714-82) or 0.25 µg of Anti-Human/Mouse T-bet eFluor® 660. This antibody has 31 citations for flow cytometry application.

Rat anti-mouse ROR gamma (t) PE (clone B2D, Catalog # 12-6981-80, eBioscience), flow cytometry, validation information from manufacturer's website. Validated using C57Bl/6 splenocytes with Anti-Mouse CD4 FITC (Product # 11-0041-82) and 0.125 µg of Rat IgG1 K Isotype Control PE (Product # 12-4301-82) or 0.125 µg of Anti-Mouse ROR gamma (t) PE. This antibody has 23 citations for flow cytometry application.

Rat anti-mouse EOMES Alexa Fluor® 488 (clone Dan11mag, Catalog # 53-4875-80, eBioscience), flow cytometry, validation information from manufacturer's website. Validated using C57Bl/6 splenocytes with Anti-Mouse NK1-1 eFluor® 450 (Product # 48-5941-82) followed by intracellular staining with 0.25 µg of Rat IgG2a K Isotype Control Alexa Fluor® 488 (Product # 53-4321-80) or 0.25 µg of Anti-Mouse EOMES Alexa Fluor® 488. This antibody has 10 citations for flow cytometry application.

Rat anti-mouse CD4 APC (clone RM4-5, Catalog # 561091, BD Biosciences), flow cytometry, validation information from manufacturer's website. This antibody has 14 citations.

Rat anti-mouse CD45 PerCP-Cyanine5.5 (clone I3/2.3, Catalog # 147705, BioLegend), flow cytometry, validation information from manufacturer's website. Validated using C57BL/6 splenocytes with CD45 (clone I3/2.3) PerCP/Cyanine5.5 or Rrat IgG2b PerCP/ Cyanine5.5 isotype control. This antibody has 3 citations.

Rat anti-mouse CD19 Alexa Fluor® 700 (clone 1D3, Catalog # 56-0193-80, eBioscience), flow cytometry, validation information from manufacturer's website. Validated using C57Bl/6 splenocytes with Anti-Human/Mouse CD45R (B220) FITC (Product #

11-0452-82) and 0.25 µg of Rat IgG2b Isotype Control APC (Product # 17-4031-82) or 0.25 µg of Anti-Mouse CD3 APC. This antibody has 41 citations for flow cytometry application.

Rat anti-mouse CD3 APC (clone 17A2, Catalog # 17-0032-80, eBioscience), flow cytometry, validation information from manufacturer's website. Validated using C57Bl/6 splenocytes with Anti-Human/Mouse CD45R (B220) FITC (Product # 11-0452-82) and 0.25 µg of Rat IgG2b Isotype Control APC (Product # 17-4031-82) or 0.25 µg of Anti-Mouse CD3 APC. This antibody has 54 citations for flow cytometry application.

Armenian hamster anti-mouse TCR gamma/delta PE (clone GL-3, Catalog # 12-5711-81, eBioscience), flow cytometry, validation information from manufacturer's website. Validated using C57BL/6 lymph node cells (preblocked with Anti-Mouse CD16/32 Purified (Product # 14-0161-82) with Anti-Mouse CD3e APC (Product # 17-0031-82) and 0.25 µg of Armenian Hamster IgG Isotype Control PE (Product # 12-4888-81) or 0.25 µg of Anti-Mouse gamma delta TCR PE. This antibody has 32 citations for flow cytometry application.

Armenian hamster anti-mouse TCR beta FITC (clone H57-597, Catalog # 11-5961-81, eBioscience), flow cytometry, validation information from manufacturer's website. Validated using BALB/c splenocytes with Anti-Mouse CD4 APC (Product # 17-0041-82) and 0.25 µg of Armenian Hamster IgG Isotype Control FITC (Product # 11-4888-81) or 0.25 µg of Anti-Mouse TCR beta FITC. This antibody has 63 citations for flow cytometry application.

Rat anti-mouse CD45 PE (clone I3/2.3, Catalog # 147711, BioLegend), flow cytometry, validation information from manufacturer's website. Validated using C57BL/6 splenocytes with CD45 (clone I3/2.3) PE or rat IgG2b PE isotype control. This antibody has 1 citation.

Rat anti-mouse CD11b APC (clone M1/70, Catalog # 101211, BioLegend), flow cytometry, validation information from manufacturer's website. Validated using C57BL/6 mouse bone marrow cells with CD11b (clone M1/70) APC or rat IgG2b, κ APC isotype control. This antibody has 135 citations.

Rat anti-mouse Ly6G FITC (clone 1A8-Ly6g, Catalog # 11-9668-80, eBioscience), flow cytometry, validation information from manufacturer's website. Validated using C57Bl/6 splenocytes with Anti-Mouse Ly-6C eFluor® 450 (Product # 48-5932-82) and 0.25 µg of Rat IgG2a K Isotype Control FITC (Product # 11-4321-42) or 0.25 µg of Anti-Mouse Ly-6G (Gr-1) FITC. This antibody has 20 citations for flow cytometry application.

Eukaryotic cell lines

Policy information about cell lines

Cell line source(s)	The IEC-6 [IEC6] (ATCC® CRL-1592™) cells were from ATCC.
Authentication	The IEC-6 cells were purchased and authenticated from ATCC, and the cell line was not authenticated by the authors.
Mycoplasma contamination	The IEC-6 cells were tested negative to mycoplasma contamination.
Commonly misidentified lines (See ICLAC register)	No commonly misidentified cell lines were used in this study.

Animals and other organisms

Policy information about studies involving animals; ARRIVE guidelines recommended for reporting animal research

Laboratory animals	All mice were purchased from the Jackson Laboratory: C57BL/6J, Ahrtm3.1Bra/J, B6.Cg-Tg(Vil1-cre)997Gum/J, B6.129P2-Lyz2tm1(cre)Ifo/J, B6.C-Tg(CMV-cre)1Cgn/J, C57BL/6-Il22tm1.1(icre)Stck/J, B6.129P2(Cg)Rorctm2Litt/J, B6.129P2-Gt(ROSA)26Sortm1(DTA)Lky/J, B6.129S-Tcrdtm1.1(cre/ERT2)Zhu/J.<br>7-day-old mice were used for experimental NEC models, and 11-day-old mice were used for endotoxemia models.<br>The White Yorkshire (Yorkshire x Landrace) sows were obtained from Oak Hill Genetics, and piglets were delivered prematurely.<br>Both male and female mice and piglets were used.
Wild animals	The study did not involve wild animals.
Field-collected samples	The study did not involve samples collected from the field.
Ethics oversight	All mouse and piglet experiments were approved by the Johns Hopkins University Animal Care and Use Committee.

Note that full information on the approval of the study protocol must also be provided in the manuscript.

Human research participants

Policy information about studies involving human research participants

Population characteristics	De-identified samples from NEC patients were collected.

Recruitment	The NEC patients who underwent surgery for NEC or at the time of stoma closure were recruited for ileal sample collection. The samples were collected in a de-identified manner via waiver of consent, and therefore the self-selection bias does not apply.
Ethics oversight	De-identified human ileal samples were collected during surgery for NEC or at the time of stoma closure, and the Office of Human Subjects Research Review Boards at Johns Hopkins University approved the collection and use of the samples for the study and waived the informed consent (IRB00094036). The IRB waived a requirement to obtain informed consent as the intestinal tissue was discarded, and was obtained during the course of a surgical procedure that was not affected by the study, and because no demographic information was collected, there was no risk to patients, and immortalized stem cells were not established.

Note that full information on the approval of the study protocol must also be provided in the manuscript.

Flow Cytometry

Plots

Confirm that:

- [x] The axis labels state the marker and fluorochrome used (e.g. CD4-FITC).
- [x] The axis scales are clearly visible. Include numbers along axes only for bottom left plot of group (a 'group' is an analysis of identical markers).
- [x] All plots are contour plots with outliers or pseudocolor plots.
- [x] A numerical value for number of cells or percentage (with statistics) is provided.

Methodology

Sample preparation	IELs were isolated from the new born mouse small intestine according to the methods of Sheridan et al56. In brief, the mesentery was removed from the freshly isolated ileum, and the bowel was then opened longitudinally and cut into 0.5cm pieces, and incubated in HBSS containing 10% fetal bovine serum, 10mM HEPES and 1mM dithioerythritol (Sigma-Aldrich) at 37° C for 20 minutes with agitation at 180 rpm. After filtration through a 70µm cell strainer, IELs were collected between the interface of 40% and 60% discontinuous Percoll in preparation for flow cytometry.<br>LP cells were isolated from the newborn mouse ileum according to the methods of Hepworth et al57. In brief, the mesentery was removed from the freshly isolated ileum, and the bowel was then opened longitudinally and cut into 1-cm pieces, and incubated in PBS containing 5% fetal bovine serum, 1mM dithioerythritol (Sigma-Aldrich) and 1mM EDTA at 37°C for 20 minutes with agitation at 180 rpm. After filtration through a 70-µm cell strainer, the remaining tissue was finely minced with scissors, and incubated in RPMI containing 2% fetal bovine serum, 0.5mg/mL collagenase/dispase (Sigma-Aldrich), and 0.02mg/mL DNase (Sigma-Aldrich) at 37°C for 40 minutes with agitation at 180 rpm. After filtration through sequential 70µm and 40µm cell strainers, the lamina propria leukocytes were collected between the interface of 40% and 60% discontinuous Percoll in preparation for flow cytometry.
Instrument	The samples were analyzed on a BD LSRII flow cytometer for ILCs and IELs, or BD Accuri™ C6 Plus flow cytometer for Th17 cells, CD45+ cells and neutrophils.
Software	FACSDiva v8.0.2 software was used to collect data from BD LSRII flow cytometer.<br>BD Accuri C6 Software was used to collect data from BD Accuri™ C6 Plus flow cytometer.<br>FlowJo™ v10.6.1, flow cytometry analysis and graphing software from FlowJo.
Cell population abundance	No cell sorting in this study.
Gating strategy	For Th17 cells, LP cells (SSC-A vs. FSC-A) were first gated for the uptake of the Live/Dead stain (boundary up to 10^4) to determine live versus dead cells, and the live cell gate was further analyzed for the expression of CD4 (boundary 2X10^3 to 10^5) and RorgtGFP (boundary 3X10^3 to 10^5).<br>for ILCs, LP cells (SSC-A vs. FSC-A) were first gated for the uptake of the Live/Dead stain (boundary up to 10^4) to determine live versus dead cells, and the live cell gate was further analyzed for the expression of hematopoietic cell lineages (CD3e, CD5, CD45R, CD11c and CD11b, boundary up to 10^3) and CD90.2(boundary 2X10^3 to 10^5). Then the expression of T-bet (ILC1, boundary 3X10^3 to 10^5), GATA-3 (ILC2, boundary 2X10^3 to 10^5) and RORgt (ILC3, boundary 2X10^3 to 10^5) was determine from the lineages- CD90+ gate.<br>For CD45+ cells and neutrophils, LP cells were first gated for singlets (FSC-H vs. FSC-A) and for the uptake of the Live/Dead stain (boundary up to 10^4) to determine live versus dead cells. The live cell gate was further analyzed for the expression of CD45 (boundary 9X10^3 to 10^6). Then the expression of CD11b (boundary 10^4 to 5X10^5) and Ly6G (boundary 10^4 to 5X10^5) was determine from the CD45+ gate.<br>For IELs, the IEL cells (SSC-A vs. FSC-A) were first gated for singlets (FSC-H vs. FSC-A), and then for the uptake of the Live/Dead stain (boundary up to 10^3) to determine live versus dead cells and the expression of CD45 (boundary 2X10^3 to to 10^5). The CD45+ gate was furthere analyzed for CD19 (boundary 2X10^3 to to 10^5) and CD3 (boundary 8X10^3 to to 10^5), and finally the CD19- CD3+ gate was analyzed for TCRgammadelta (boundary 6X10^2 to to 10^5) and TCRbeta (boundary 2X10^3 to to 10^5) expression.

- [x] Tick this box to confirm that a figure exemplifying the gating strategy is provided in the Supplementary Information.
